# The Expressions of the Immunity- and Muscle Development-Related Genes of 40-Day-Old Broilers Are Promoted in Response to the *In Ovo* and Dietary Supplemental Administration of Calcidiol in Conjunction with the *In Ovo* Administration of Marek’s Disease Vaccine †

**DOI:** 10.3390/ani15010010

**Published:** 2024-12-24

**Authors:** Seyed Abolghasem Fatemi, April Waguespack Levy, Edgar David Peebles

**Affiliations:** 1 Department of Poultry Science, Mississippi State University, MS 39762, USA; d.peebles@msstate.edu; 2 DSM Nutritional Products, Parsippany, NJ 07054, USA; april.levy@dsm.com

**Keywords:** calcidiol, immunity, *in ovo* injection, Marek’s disease vaccine, muscle development

## Abstract

Effects of *in ovo* and dietary sources of calcidiol (25(OH)D_3_), combined with Marek’s disease vaccine (MDV), on the expression of genes involved with the antioxidant activity, muscle deposition, and immunity in the pectoralis major (P. major) muscle and spleen of 40 d of age (doa) broilers were investigated. The *in ovo* treatments were as follows: (1) non-injected; (2) the injection of 50 μL of commercial MDV, (3) MDV + 1.2, or (4) 2.4 μg of 25(OH)D_3_. All birds received either a commercial diet containing no supplemental 25(OH)D_3_ (control) or the same diet supplemented with an additional 69 µg of 25(OH)D_3_ per kg of feed (Hy-D diet). At 40 doa, the pectoralis major (P. major) muscle and spleen of 48 birds (six replicates per diet x *in ovo* treatment combination) were collected. When compared to un-supplemented commercial diet-fed birds, in birds that were fed the Hy-D diet, the expression of the TGF-β4 gene in the spleen and P. major muscle, and the *GSH-P1*, *GSH-P7*, *SOD2*, *MyoG*, *MyoD1*, and *Pax3* genes in the P. major muscle were up-regulated, whereas the expression of the *IL-1β*, *IL-8*, and *CYP24A1* genes in the spleen and P. major muscle were down-regulated. Nevertheless, birds that received any of the *in ovo* injection doses of 25(OH)D_3_ exhibited a higher expression of the *IL-10*, *TGF-β4*, and *CYP27B1* genes in the spleen and P. major muscle. Furthermore, in comparison to the MDV-injected control group, the *CAT*, *MyoD1*, and *Pax3* genes in the P. major muscle were up-regulated, and the expression of the *INF-γ*, *IL-1β* and *CYP24A1* genes in the spleen and the *IL-8*, and *IL-1β* genes in the P. major muscle were down-regulated. In conclusion, a significant improvement in the expression of genes responsible for enzymatic antioxidant activity, protein synthesis, and inflammatory reactions in 40-day-old broilers occurred in response to *in ovo* and dietary supplemental 25(OH)D_3_, and supplemental 25OHD_3_ provided via either route was used to enhance the expression of genes linked to vitamin D activity (*CYP27B1*, *CYP24A1*).

## Simple Summary

Calcidiol (25(OH)D_3_) has previously been shown to exhibit promising results concerning the expression of genes linked to decreased inflammatory reactions as well as increased muscle deposition in broilers. The purpose of the current study was to identify the impacts of supplemental 25(OH)D_3_ administered dietarily or by *in ovo* injection in conjunction with the *in ovo* delivery of Marek’s Disease vaccine (MVD), on the expression of genes in the spleen and pectoralis major (P. major) of 40 days of age Ross 708 broilers. Although the dietary, rather than the in ovo source of 25(OH)D_3_, had a greater potency, when compared to MDV alone, both sources of 25(OH)D_3_ enhanced the expression of genes that function in adaptive immunity in both the spleen and P. major. Furthermore, they also potentiated enzymatic antioxidant activity and muscle deposition. In conclusion, supplemental 25(OH)D_3_, delivered either through the diet or by *in ovo* injection, can be used to promote the expression of genes involved in the growth and immunity of 40-day-old broilers that received an MDV vaccination by *in ovo* injection.

## 1. Introduction

Intensive genetic selection in broilers that took place between 1957 and 2005 has resulted in a rapid increase in the yield of the pectoralis major (**P. major**) and pectoralis minor (**P. minor**) muscles by approximately 80% and 33%, respectively. Additionally, the feed conversion ratio (**FCR**) during that same period of time has decreased annually by 2.55%, which has accounted for a cumulative 50% reduction in the FCR [1]. Although beneficial breast yield results have been observed over 50 years of commercial quantitative genetic selection, serious abnormalities have also been observed in breast filets. For instance, white striping (**WS**) and woody breast (**WB**) conditions have been recently observed in modern broilers [2,3]. Approximately 90% of the P. major in broilers exhibit some level of WS and WB, which has caused an estimated USD 200 million to USD 1 billion in economic losses in the US poultry industry [4]. Furthermore, both WS and WB are commonly not only associated with immune-related cell infiltration, but also to an increase in oxidative reactions, fibrosis, and lipidosis in muscle tissue [5,6]. Besides physiological and morphological changes in WB filets, several changes have taken place at molecular levels. Whole genome sequencing analysis has revealed that genetically regulated metabolic pathways associated with protein synthesis were down-regulated and that those linked to inflammation and oxidative or endoplasmic reticulum stresses were upregulated [7,8]. These results indicate that an enhancement of the expression of genes involved in protein synthesis and reduced oxidative stress can be beneficial in lowering the incidence of WB.

The technology of *in ovo* injection has been used for many decades for the early vaccination of broilers against infectious diseases such as Marek’s disease [9,10]. In addition to vaccination, various nutrients including vitamins and minerals have been administered *in ovo* in order to promote the immunity and growth of commercial broilers [11,12,13,14]. The combined effects of Marek’s disease vaccine (**MDV**) with the second metabolite of vitamin D_3_, calcidiol (**25(OH)D_3_**), have been investigated. Fatemi et al. [15] reported that the MDV can be successfully combined with various doses of 25(OH)D_3_, ranging from 0.6 to 2.4 μg, with a minimal death (5%) of MDV-carrying cells. Calcidiol has been previously shown to exhibit promising results on broiler performance when administered by *in ovo* injection in a range between 0.6 and 2.4 μg or through the diet at 69 μg/kg throughout a 42 doa rearing period. These included positive effects on bone development [16,17,18,19], early posthatch and long-term live performance variables (BW, BWG, FCR) [20,21,22,23,24], breast [21,22,23,24,25,26] and leg meat [24] yield, serum IgG and nitric oxide representative inflammatory immune responses [27,28,29,30,31,32,33,34], serum and intestinal enzymatic (glutathione S-transferase and superoxide dismutase) and non-enzymatic antioxidant (malonaldehyde) activities [35], and intestinal histomorphology [25,35,36] when broilers were raised under either commercial or stressful conditions, such as a coccidial infection. It is suggested that the realized improvements in response to 25(OH)D_3_ are associated with its longer half-life [37,38] as well as a cascade in the expression of genes, which includes an increase in vitamin D receptors (***VDR***) and production of the active form of vitamin D_3_, *1,25-dihydroxyvitamin D_3_* (**1,25-(OH)_2_-D_3_**) [26,28,30,39,40]. More recently, as compared to the MDV alone, the *in ovo* injection of the MDV in combination with both dietary (69 µg) and *in ovo* (1.2 and 2.4 µg) sources of 25(OH)D_3_, has been shown to result in an increase in body weight (**BW**), BW gain (**BWG**) breast and leg meat yield [41], and a decrease in the inflammatory reaction [42] in both 14- and 40-day of age (**doa**) broilers. In addition, villus height and villus height to crypt depth were increased, and crypt depth was deceased in those broilers [42] in response to both 25(OH)D_3_ sources. The authors suggested that the abovementioned improvements were due to the stimulation of genes involved in vitamin D_3_ activity as well as genes linked to muscle deposition and immunity. However, this hypothesis has not been previously tested in mature broilers that received the MDV in conjunction with *in ovo* and dietary supplemental 25(OH)D_3._ Therefore, the objective of the current study was to investigate the change in the expression of genes involved in antioxidant activity, anti-inflammatory and pro-inflammatory responses, and muscle growth in 40 doa Ross 708 broilers, having received an *in ovo* injection of the MDV along with dietary and *in ovo* sources of supplemental 25(OH)D_3_.

## 2. Materials and Methods

### 2.1. Experimental Condition

The incubational regime as well as the experimental parameters in this study were as described by Fatemi et al. [39,41]. At 18 days of incubation (**doi**), 4 *in ovo* injection treatments were applied. These included a 50 μL volume of commercial MDV (control) or commercial MDV in combination with either 1.2 (**MDV + 25(OH)D_3_-1.2**) or 2.4 (**MDV + 25(OH)D_3_-2.4**) μg of 25OHD_3_. A non-injected treatment group was also included as another control group. All injections were administered using a Zoetis Inovoject m machine (Zoetis Animal Health, Research Triangle Park, NC, USA). A water-soluble form of 25(OH)D_3_ (ROVIMIX^®^ Hy-D^®^ 1.25%) was used, which was provided by DSM Nutritional Products (DSM Nutritional Products Inc. Parsippany, NJ, USA). All injection solutions were freshly prepared as specified by Fatemi et al. [15,39]. Chicks were pulled from the hatcher and feather-sexed at 502 h of incubation, or 21 doi. Thirteen male broilers were randomly placed at a 0.62 m^2^/bird stocking density in each of 48 floor pens. The birds in 24 of the pens received a control commercial diet containing 250 IU of vitamin D_3_ /kg of feed without any supplemental 25(OH)D_3_ (control). Birds in the other 24 pens received a control diet containing an additional 69 μg of 25(OH)D_3_ /kg of feed (**Hy-D diet**) throughout 40 doa. All diets were formulated in accordance with Ross 708 commercial guidelines [43], and the experimental dietary compositions for the starter (0 to 14 doa), grower (15 to 28 doa), and finisher (29 to 40 doa) grow out phases were as described by Fatemi et al. [41]. The actual levels of vitamin D_3_ and 25(OH)D_3_ in both the control and Hy-D diets were tested in order to maintain feed formulation accuracy. Test results showed that there was a maximum of 10% difference between the calculated and actual values [41]. Forty doa was chosen for sample collection according to previous studies, showing significant changes in the immunological, morphological variables [27,28,32,34,42], meat yield [22,23,24,25,26,34,41] and the presence of WB [23,41] in response to *in ovo* or dietary 25(OH)D_3_.

### 2.2. Gene Expression Analysis

At 40 doa, 6 replicate birds in each of 8 treatment groups (2 dietary × 4 *in ovo* treatments; 48 total) were selected and individually weighed. From the same bird, approximately 5 to 10 g of the medial apex of the P. major and complete spleen were collected. All tissues were frozen in cryogenic tubes in liquid nitrogen and then stored at −80 °C until RNA isolation. RNA isolation and purification were performed using the method described by Fatemi et al. [28]. The house-keeping genes for this study were glyceraldehyde-3-phosphate dehydrogenase (***GAPDH***) and 10 other target genes functioning in vitamin D_3_ activity [1α-hydroxylase (***CYP27B1***), 24-hydroxylase (***CYP24A1)***, and *VDR*], innate immunity [toll like receptor *(**TLR***)*-3*, *TLR-7*, and *TLR-21*], pro-inflammatory response [interferon (***INF***)-*α*, *INF-β*, and *INF-γ*, interleukins (***IL***)-*8*, *IL-10*, and *IL-1β*], anti-inflammatory response [*IL-10*, chicken transforming growth factor-beta 4 (***TGF-β4***)], antioxidant capacity [catalase (***CAT***), glutathione S-transferase-P1 (***GSH-P1***), glutathione S-transferase-P2 (***GSH-P2***), superoxide dismutase-1 (***SOD1***), and superoxide dismutase-2 (***SOD2***)], muscle development [paired box domain 3 (***Pax3***), myogenin (***MyoG***), myogenic regulatory transcription factor 4 (***Mrf4***), and myogenic differentiation proteins (***MyoD1***)]. The forward and reverse primers have been described by Fatemi et al. [40], Karpala et al. [44], Gimeno et al. [45], Tang et al. [46], and Yin et al. [47]. The immunity as well as vitamin D_3_ activity genes were identified in the spleen tissue, while antioxidant activity, anti-inflammatory and pro-inflammatory responses, muscle development, and vitamin D activity were determined in the P. major. Real time PCR reactions took place in duplicate in a total volume of 20 μL, which contained 2 μL of cDNA, 0.6 μL of forward and reverse primers (10 μM) for each gene, 6.8 μL of double distilled water (ddH_2_O), and 5 μL of SYBR Green premix. Melting curve analysis and target gene normalization were performed in accordance with the procedure described by Fatemi et al. [40]. A comparative 2^−ΔΔCT^ method was used to calculate fold-change differences in target gene expression relative to the endogenous control.

### 2.3. Statistical Analysis

A randomized block was the statistical experimental design, and the experimental unit was floor pen. One bird from each of the 4 *in ovo* treatments within each of 6 replicate pens per dietary treatment (2 dietary × 4 *in ovo* treatments × 6 pens; 48 total) were used. The normal distribution of the data was performed using the Shapiro–Wilk test at α = 0.05 for all endogenous and target genes, and all genes were observed to be normally distributed. All gene expression data were analyzed as a 2-way analysis of variance with a 4 × 2 factorial arrangement of treatments (4 *in ovo* injection and 2 dietary treatments) in order to obtain both significant main and interaction treatment effects. The abovementioned data were analyzed using SAS 9.4© [48] with a general linear mixed models’ procedure (PROC GLIMMIX), and *p* ≤ 0.05 was set for treatment differences. Noteworthy trends were set at *p* < 0.06. Fisher’s protected LSD was used for treatment means separations [49] in order to realize more notable treatment differences.

## 3. Results

### 3.1. Immunity

There were no significant interactive treatment effects for immunity-related genes in either the spleen and P. major. However, in the spleen, the *INF-γ* (*p* = 0.001) and *IL-1β* (*p* = 0.001) genes were down-regulated in birds that were injected with any of the 25OHD_3_ doses when compared to the non-injected or MDV alone-injected control treatment groups. Additionally, the expression of the *IL-1β* (*p* = 0.042) and *IL-8* (*p* = 0.021) genes decreased and that of the *IL-10* (*p* = 0.003) and *TGF-β4* (*p* = 0.001) genes increased in birds fed Hy-D rather than commercial diets. Furthermore, the *IL-10* (*p* = 0.003) and *TGF-β4* (*p* = 0.009) genes were up-regulated in the MDV+25(OH)D_3_-2.4 treatment group as compared to both control treatment groups, while the expression of those genes was lower in the MDV alone treatment group than those belonging to the MDV + 25(OH)D_3_-1.2 treatment group (Table 1). In the P. major, broilers fed Hy-D-fed diets exhibited a lower expression of the *IL-1β* gene (*p* < 0.001) and a higher expression of the *TGF-β4* gene (*p* < 0.001) in comparison to broilers that were fed commercial diets. Additionally, when compared to either control treatment group, down-regulation of the *IL-1β* (*p* = 0.001) and *IL-8* (*p* = 0.008) genes was observed in birds that belonged to any treatment groups containing 25(OH)D_3_. Furthermore, compared to the non-injected treatment group, upregulation of the *TGF-β4 gene* (*p* = 0.05) was observed in the MDV + 25(OH)D_3_-1.2 treatment group (Table 2).

### 3.2. Antioxidant Activity

Similar to the immunity-related genes, only the main treatment effects were significant for antioxidant activity-related genes in the P. major. In comparison to unsupplemented commercial diets, the expressions of the *SOD2* (*p* = 0.001), *GSH-P1* (*p* = 0.001), and *GSH-P2* (*p* = 0.024) genes were up-regulated in response to the feeding of supplemental Hy-D diets. In addition, birds that received any dose of *in ovo*-injected 25(OH)D_3_ exhibited a higher expression of the *CAT* (*p* = 0.049) gene in comparison to those birds that belonged to either the non-injected or MDV-injected treatment groups. Furthermore, the expression of the *SOD2* gene tended (*p* = 0.059) to be higher in birds that belonged to the MDV + 25(OH)D_3_-2.1 treatment group relative to those in the non-injected and MDV-injected treatment groups (Table 3).

### 3.3. Muscle Development

The *MYF 4* gene, which is linked to muscle deposition, was not affected by the *in ovo* or dietary treatments. However, as compared to the feeding of the unsupplemented commercial diet, up-regulation of the *MyoD1* (*p* = 0.006), *MyoG* (*p* < 0.001), and *Pax3* (*p* = 0.013) genes occurred in response to the feeding of the supplemental Hy-D diet. Furthermore, when compared to the non-injected or MDV-injected treatments, increases in the expressions of the *MyoD1* (*p* = 0.006) and *Pax3* (*p* = 0.013) genes were observed in birds that belonged to MDV + 25(OH)D_3_-1.2 and MDV + 25(OH)D_3_-2.4 treatments (Table 4).

### 3.4. Vitamin D Activity

The results for the vitamin D activity-related genes are presented in Table 5. There was a significant interactive effect between the *in ovo* and dietary treatments for the *CYP27B1* (*p* = 0.010) gene in the spleen. In birds fed Hy-D diets, a higher expression of the *CYP27B1* gene was observed when they received an *in ovo* injection of 1.2 μg of 25(OH)D_3_ in comparison to those that received any of the other *in ovo* injection treatments. Also, in birds fed Hy-D diets, the *in ovo* injection of 2.4 μg of 25(OH)D_3_ up-regulated expressions of the *CYP27B1* gene in comparison to those that belonged to the non-injected treatment group. However, in birds fed the unsupplemented commercial diet, there was no significant difference among *in ovo* injection treatments. Additionally, in birds fed the Hy-D diet, the splenic expression of the *CYP27B1* gene was significantly higher when they received the MDV + 25(OH)D_3_-1.2 *in ovo* treatment rather than the other *in ovo* treatments. In the P. major, the expression of the *CYP27B1* gene was significantly (*p* = 0.050) greater in birds that were fed the Hy-D diet and was significantly (*p* < 0.001) greater in those that were *in ovo*-injected with either dose of 25(OH)D_3_ when compared to those that belonged to the non-injected and MDV-injected treatment groups. In both the spleen and P. major, feeding of the Hy-D diet rather than the commercial diet reduced the expression of the *CYP24A1* gene. Furthermore, splenic expression of the *CYP24A1* gene was greater in birds belonging to the 25(OH)D_3_ *in ovo*-injected treatment group than in those in both control treatment groups, and a higher expression of the *CYP24A1* gene was observed in birds belonging to the non-injected treatment group in comparison to those in the 25(OH)D_3_-1.2 and MDV + 25(OH)D_3_-2.4 treatment groups (Table 5).

## 4. Discussion

The results of the current study showed that there were no changes in the expressions of genes linked to the innate immune (*TLR-3*, *TLR-7*, and *TLR-21*) response of 40-day-old broilers. The innate immunity-related genes tested in this study are elicited in the first line of detection and subsequent defense against infectious viruses [50], bacteria [51], and protozoa [28]. An increase in the expression of the TLR family of genes in primary or secondary immune tissues can be an indication of enteritic pathogenic infections [52]. No viral or bacterial infection was detected in the live and dead birds between 0 and 40 doa in the companion study in which similar *in ovo* and dietary treatments were used [41]. Other immune-related genes were categorized into two types. Pro-inflammatory response genes [*IL-1β*, *IL-8*, and *INF-γ*] were the first type categorized, and then anti-inflammatory response-type genes [*IL-10* and *TGF-β4*] were secondarily categorized. In the current study, the administration of dietary and *in ovo* sources of 25(OH)D_3_ in combination with the *in ovo* administration of the MDV, increased the expression of anti-inflammatory response genes and decreased the expression of pro-inflammatory response genes in the breast filets and spleens of broilers. Similar results were also reported in the companion study when an improvement in anti-inflammatory and pro-inflammatory response genes were observed in 1.2 and 2.4 μg of *in ovo* or at 69 μg/kg dietary of 25(OH)D_3_ combined with *in ovo* MDV.

Pro-inflammatory response genes are a group of genes that mainly stimulate the recruitment of adaptive immune-related cells to the site of an inflection, lead to the destruction of infectious organisms and infected host cells, and produce specific antibodies against various types of pathogens [53,54]. On the other hand, anti-inflammatory response genes suppress immune reactions by reducing the production of active immune cells that subsequently result in a decrease in acute and chronic inflammation [54,55,56,57]. It is worth mentioning that an increase in an inflammatory response is not always unfavorable. Particular pro-inflammatory responses that are linked to humoral immunity or anti-body production [*INF-α* and *INF-β*] have been shown to be quickly elevated after vaccination [58,59,60] or during the first week of life when the immune system of chicks is underdeveloped [61,62]. In addition, although rapid inflammatory and immune cell responses are beneficial in reducing an individual’s exposure to enteric pathogens, chronic inflammatory reactions can be detrimental [31,32,33,34]. A chronic immune reaction can negatively affect growth [23,24,28,29,40,41,42], muscle deposition [23,24,28,29,40,41,42], and oxidative stress resistance [63] in broilers. When both dietary (69 μg/kg feed) [32,34] and *in ovo* (2.4 μg) [28,39,40] sources of 25(OH)D_3_ were individually administered in normal or coccidiosis-infected poultry species, positive effects have been observed on the expressions of anti-inflammatory (*IL-1β*) and pro-inflammatory response (*IL-10* and *TGF-β4*) genes. An improvement in inflammatory response gene expression in response to 25(OH)D_3_ sources has been shown to be positively correlated with intestinal histomorphology, growth performance, and meat yield variables [23,24,27,31,32,33,39,40]. The improvement in immunity-related genes in response to 25(OH)D_3_ is correlated with an increase in CYP27B1 and a decrease in CYP24A1 expression [27,39,40]. In this study, the up-regulation of the CYP27B1 gene and down-regulation of the CYP24A1 gene were observed in breast muscle in response to both 25(OH)D_3_ sources may be the reason for the effective modulation of the inflammatory response genes. Therefore, both 25(OH)D_3_ sources can be used to lower inflammatory reactions and improve chronic immunity in the breast muscle and spleen tissues of mature broilers.

Hydrogen peroxide (**H_2_O_2_**) can damage cellular components, resulting in the production of neutral molecules. However, the catabolism of H_2_O_2_ is facilitated by redox active metal ions such as cooper (**Cu**), iron (**Fe**), and manganese (**Mn**) in association with antioxidant enzymes including CAT, glutathione S-transferase (**GSH**), and glutathione peroxidase (**GPx**) [64]. In the chicken, SOD in conjunction with Cu, Fe, and Zn has been shown to increase free radical detoxification by converting superoxide radicals into oxygen and H_2_O_2_ [65,66] that can subsequently be decomposed into water by the action of CAT and GPx [65,66,67,68,69]. It is well demonstrated that the expression of *SOD* and *GSH* families and *CAT* genes are down-regulated in WB filets, indicating an increase in oxidative reactivity as the result of a suppression in antioxidant capacity [66,67,68,69]. Furthermore, significantly higher reactive oxygen species in association with a lower antioxidant defense system have been observed in WB filets [70,71]. In addition, a strong association has been observed between oxidative stress or the suppression of non-enzymatic and enzymatic activities with an increase in pro-inflammatory response genes, which leads to an increase in systemic chronic inflammation [72]. In this study, the aforementioned genes were up-regulated by either dietary or *in ovo* 25(OH)D_3_ sources. In particular, the positive effects resulting from the use of 69 µg of supplemental dietary 25(OH)D_3_ indicates that these sources can be used to lower negative effects in WB filets due to oxidative reactions. However, further research is needed to determine the precise relationship between dietary or *in ovo* 25(OH)D_3_ sources with enzymatic antioxidant capacities in WB filets.

Major genes that are associated with the stimulation of muscle fiber synthesis include *MyoD1*, *MyoG*, *Mrf4*, and myogenic factor 5 (***Myf5***) in both prenatal [73] and postnatal periods [40,74,75]. In particular, the stimulation of muscle fiber synthesis by these genes has been observed during those periods when various vitamin D sources were administrated by *in ovo* or dietary supplementation. Chicken myoblast proliferation has been shown to be modulated by the *Myf5* and *MyoD* genes [76,77], while the onset of muscle differentiation is mediated by the *MyoG* gene through its role in cell lineage specification during myogenesis [76,78]. Also, a decrease in the expression of the *MyoD1* and *Myf5* genes was reported when the *CYP24A1* expression was up-regulated, resulting in the inhibition of myogenesis during early chicken embryo development [73]. Not only are the aforementioned genes involved in muscle development, but their increased expression has also been shown to be significantly correlated with the BW and breast and leg meat yield of broilers. Genxi et al. [79] reported that higher P. major muscle mass, BW, and BWG were observed in broilers when the expression of the MyoG and Myf5 genes were up-regulated. These results indicate that the crucial role of chicken myogenic regulatory factor (***MRF***) genes is in not only the muscle deposition but also the overall post-hatch performance of broilers. Furthermore, posthatch muscle fiber proliferation and repair are directly linked to MRF gene activity and the quantity and size of satellite cells (**SC**s) [80]. It is well documented that an increase in the number and size of SCs results in an increase in breast meat yield in association with an increase in protein synthesis [22]. These relationships have been observed to be associated with the expression of paired box domain (***PAX***) genes such as *Pax3* and *Pax7* [81]. In addition, increased SC-derived myoblast proliferation has been observed when *Pax3* gene expression was up-regulated [82,83]. Moreover, a positive linear correlation between *MyoD1* and *MyoG* gene activity during SC proliferation has been noted in poultry [84]. An increase in the rate of protein synthesis and *Pax3* and *Pax7* gene expression, leading to an increase in SC size and number, were observed in Ross 308 broilers at 42 doa in response to 69 µg of dietary 25(OH)D_3_/kg of feed [22]. More recently, in a companion study conducted by Fatemi et al. [40], improvements in live performance and meat yield were reported when the expressions of *MyoD1*, *MyoG*, and *Myf4* (MRF genes) as well as *Pax3* (an SC-related gene) were up-regulated in 14 doa broilers that were fed commercial diets supplemented with 69 µg of 25(OH)D_3_/kg of feed. Similar results were observed in this study, indicating that both 25(OH)D_3_ sources are capable of increasing the rates of SC cell proliferation and muscle deposition. However, further research is needed to determine possible morphological changes in the muscle fibers of boilers that received either25(OH)D_3_ source in conjunction with the *in ovo* administration of the MDV.

An enhancement in the expression of genes involved in vitamin D activity could be a logical basis for improvements in the antioxidant activity-, immunity-, and growth-related responses of broilers to either source of 25(OH)D_3_. Vitamin D_3_ undergoes 2 hydroxylation steps to become an active hormone. The first hydroxylation takes place in the hepatic cell where vitamin D_3_ is converted to 25(OH)D_3_ by 25-hydroxylase enzyme. It is then subsequently converted to the active hormone, 1,25-dihydroxycholecalciferol [**1,25(OH)_2_ D_3_**], by the CYP27B1 enzyme in renal cells [85]. However, 25(OH)D_3_ can also be converted to the inactive form of vitamin D_3_, 24,25-dihydroxycholecalciferol [24,25(OH)_2_ D_3_], by the action of the CYP24A1 enzyme [85,86]. In the chicken, the CYP27B1 and CYP24A1 genes are highly expressed in the kidney, intestine, leg and breast muscle, and immune cells [87], and their expressions have been further found in low amounts in bone tissue [88]. All the functions of vitamin D_3_ are conducted by 24,25(OH)_2_. Therefore, it is responsible for the increase in the expression of the CYP27B1 and the decrease in the expression of the CYP24A1 genes attributed to the two vitamin D_3_ sources used in this study. In broilers subjected to a coccidial infection for 2 weeks, increases in their BW, breast meat yield, and anti-inflammatory response genes (IL-10 and TGF-β4), and reductions in an inflammatory indicator (α-1-acid glycoprotein) and pro-inflammatory gene (IL-6) in their serum were reported when they were injected with 2.4 µg of 25(OH)D_3_ [28]. When compared to non-injected and diluent-injected control groups in the same report, the expression of the CYP27B1 gene was up-regulated while the expression of the CYP24A1 gene tended to be lower [28]. Furthermore, when compared to a non-injected control group, an enhancement in splenic gene expression linked to innate and humoral immunity was reported in MDV *in ovo*-injected hatchlings that were concomitantly *in ovo*-injected with either 1.2 or 2.4 µg of 25(OH)D_3_. This result was observed to be associated with a higher expression of the *CYP27B1* gene and a lower expression of the *CYP24A1* gene [42]. More recently, an up-regulated expression of the *CYP27B1* gene and a down-regulated expression of the *CYP24A1* gene were associated with enhancements of skeletal muscle development-, antioxidant activity-, and immunity-related genes in the P. major and splenic tissue of 14 doa broilers [40]. An increase in protein synthesis and breast meat yield in association with the enhancement of vitamin D_3_ activity-related genes in response to supplemental dietary 25(OH)D_3_ has been previously reported [26,89]. These findings demonstrate that the promising positive results for various physiological functions in response to *in ovo* and dietary sources of 25(OH)D_3_ may be due to an enhancement in the expression of genes linked to the activation and inactivation of 1,25(OH)_2_D_3_.

## 5. Conclusions

In conclusion, these findings revealed that both sources of 25(OH)D_3_, in particular the dietary source of 25(OH)D_3_, promoted the expression of genes associated with the antioxidant defense, muscle synthesis, and immune systems of mature Ross 708 broilers. This improvement was found to be linked to the increased activity of vitamin D_3_ genes in tissues where *CYP27B1* converts 25(OH)D_3_ to the active form of vitamin D. A decrease in the activity of the *CYP24A1* gene, which converts 25(OH)D_3_ to the inactive form of vitamin D, may result in an increase in the functionality of both 25(OH)D_3_ sources in spleen and breast tissue. Therefore, it is recommended that 25(OH)D_3_ be administered either by *in ovo* injection at a dose ranging between 1.2 and 2.4 µg, or by delivering 69 µg of supplemental 25(OH)D_3_/kg of feed in order to stimulate the expression of genes linked to skeletal muscle synthesis as well as immunity in broilers having received the MDV by *in ovo* injection.

## Figures and Tables

**Table 1:** Influence of non-injected; Marek’s disease vaccine (**MDV**)-injected; and MDV treatments containing various doses of calcidiol [**25(OH)D_3_**]; and diets supplemented with or without 25(OH)D_3_ on the fold change expression of immune-related genes in the spleen of broilers at 40 days of age (**doa**).

Treatment		N ^7^	TLR-3 ^1^	TLR-7 ^1^	TLR-21 ^1^	INF-α ^1^	INF-β ^1^	INF-γ ^1^	IL-1β ^1^	IL-8 ^1^	IL-10 ^1^	TGF-β4 ^1^
*In ovo* injection												
	Non-injected ^2^	12	1.4	1.3	1.9	1.9	2.9	4.1 ^a^	1.1 ^a^	1.9	1.1 ^bc^	1.3 ^bc^
	MDV ^3^	12	1.1	1.3	1.8	1.5	2.2	4.4 ^a^	1.1 ^a^	1.4	0.9 ^c^	1.1 ^c^
	MDV + 25OHD_3_-1.2 ^4^	12	0.7	1.0	1.8	1.8	2.2	1.7 ^b^	0.5 ^b^	0.7	1.7 ^ab^	1.9 ^ab^
	MDV + 25OHD_3_-2.4 ^5^	12	0.8	1.4	1.2	1.4	2.5	1.0 ^b^	0.4 ^b^	0.9	2.1 ^a^	2.1 ^a^
	SEM		0.30	0.59	5.1	0.55	0.71	0.86	0.20	0.61	0.34	0.31
Diet												
	Commercial	24	1.1	1.5	2.0	1.9	2.5	2.9	0.9 ^a^	1.8 ^a^	1.1 ^b^	1.2 ^b^
	Hy-D ^6^	24	1.0	1.0	1.4	1.4	2.5	2.1	0.6 ^b^	0.7 ^b^	1.8 ^a^	2.0 ^a^
	SEM		0.21	0.43	0.36	0.39	0.50	0.51	0.14	0.44	0.24	0.22
			*p*-value									
*In ovo*			0.104	0.945	0.552	0.785	0.675	0.001	0.001	0.219	0.003	0.009
Diet			0.681	0.256	0.117	0.187	0.979	0.545	0.042	0.021	0.003	0.001
*In ovo* x Diet			0.691	0.298	0.724	0.706	0.174	0.358	0.455	0.897	0.435	0.618

^a–c^ Treatment means within the same column within effect with no common superscripts are significantly different (*p* ≤ 0.05). ^1^ Toll-like receptor-3; toll-like receptor-7; toll-like receptor-21; interferon-*α*; interferon-β; interferon-γ; interleukin-1β; interleukin-8; interleukin-10; and transforming growth factor-beta 4; ^2^ Embryos that were not injected with a solution. ^3^ Embryos injected with the commercial diluent in conjunction with Marek’s disease vaccine (**MDV**; turkey herpesvirus) at 18 d of incubation (**doi**). ^4^ Embryos injected with MDV containing 1.2 μg of 25OHD_3_ at 18 doi. ^5^ Embryos injected with MDV containing 2.4 μg of 25OHD_3_ at 18 doi. ^6^ A diet supplemented with 2760 IU/kg 25OHD_3_ throughout the rearing period. ^7^ Number of replications per treatment.

**Table 2:** Influence of noninjected; Marek’s disease vaccine (**MDV**)-injected; and MDV treatments containing various doses of calcidiol [**25(OH)D_3_**], and diets supplemented with or without 25(OH)D_3_ on the fold change expression of immune-related genes in the pectoralis major muscle of broilers at 40 days of age (**doa**).

Treatment		N ^7^	INF-γ ^1^	IL-1β ^1^	IL-8 ^1^	TGF-β4 ^1^
*In ovo* injection						
	Non-injected ^2^	12	2.1	1.4 ^a^	1.4 ^a^	0.9
	MDV ^3^	12	1.8	1.6 ^a^	1.2 ^a^	1.1
	MDV + 25OHD_3_-1.2 ^4^	12	1.1	0.8 ^b^	0.6 ^b^	1.6
	MDV + 25OHD_3_-2.4 ^5^	12	0.9	0.6 ^b^	0.5 ^b^	1.8
	SEM		0.61	0.26	0.29	0.26
Diet						
	Commercial	24	1.5	1.6 ^a^	1.1 ^a^	0.6 ^b^
	Hy-D ^6^	24	1.4	0.7 ^b^	0.7 ^b^	2.0 ^a^
	SEM		0.43	0.17	0.21	0.26
			*p*-value			
*In ovo*			0.152	0.001	0.008	0.050
Diet			0.737	<0.001	0.119	<0.001
*In ovo* x Diet			0.916	0.116	0.276	0.276

^a–b^ Treatment means within the same column within effect with no common superscripts are significantly different (*p* ≤ 0.05). ^1^ Interferon-γ; interleukin-1β; interleukin-8; and interleukin-10. ^2^ Embryos that were not injected with a solution. ^3^ Embryos injected with the commercial diluent in conjunction with Marek’s disease vaccine (**MDV**; turkey herpesvirus) at 18 d of incubation (**doi**). ^4^ Embryos injected with MDV containing 1.2 μg of 25OHD_3_ at 18 doi. ^5^ Embryos injected with MDV containing 2.4 μg of 25OHD_3_ at 18 doi. ^6^ A diet supplemented with 2760 IU/kg 25OHD_3_ throughout the rearing period. ^7^ Number of replications per treatment.

**Table 3:** Influence of noninjected; Marek’s disease vaccine (**MDV**)-injected; and MDV treatments containing various doses of calcidiol [**25(OH)D_3_**], and diets supplemented with or without 25(OH)D_3_ on the fold change expression of antioxidant activity-related genes in the pectoralis major muscle of broilers at 40 days of age (**doa**).

Treatment		N ^7^	SOD1 ^1^	SOD2 ^1^	GSH-P1 ^1^	GSH-P7 ^1^	CAT ^1^
*In ovo* injection							
	Non-injected ^2^	12	1.4	1.0	1.6	1.7	1.0 ^b^
	MDV ^3^	12	1.0	1.1	1.3	1.1	0.9 ^b^
	MDV + 25OHD_3_-1.2 ^4^	12	1.5	2.1 ^a^	1.4	2.2	2.1 ^a^
	MDV + 25OHD_3_-2.4 ^5^	12	1.6	1.5 ^ab^	1.5	2.0	2.4 ^a^
	SEM		0.68	0.44	0.56	0.72	0.47
Diet							
	Commercial	24	1.1	0.8 ^b^	0.7 ^b^	0.9 ^b^	1.2
	Hy-D ^6^	24	1.7	2.0 ^a^	2.2 ^a^	2.6 ^a^	1.8
	SEM		0.48	0.31	0.40	0.82	0.41
			*p*-value				
*In ovo*			0.840	0.059	0.914	0.727	0.049
Diet			0.235	0.001	0.001	0.024	0.098
*In ovo* x Diet			0.428	0.432	0.714	0.574	0.994

^a,b^ Treatment means within the same column within effect with no common superscripts are significantly different (*p* ≤ 0.05). ^1^ Superoxide dismutase-1; superoxide dismutase-2; glutathione S-transferase-P1; glutathione S-transferase-P2; and catalase. ^2^ Embryos that were not injected with a solution. ^3^ Embryos injected with the commercial diluent in conjunction with Marek’s disease vaccine (**MDV**; turkey herpesvirus) at 18 d of incubation (**doi**). ^4^ Embryos injected with MDV containing 1.2 μg of 25OHD_3_ at 18 doi. ^5^ Embryos injected with MDV containing 2.4 μg of 25OHD_3_ at 18 doi. ^6^ A diet supplemented with 2760 IU/kg 25OHD_3_ throughout the rearing period. ^7^ Number of replications per treatment.

**Table 4:** Influence of noninjected; Marek’s disease vaccine (**MDV**)-injected; and MDV treatments containing various doses of calcidiol [**25(OH)D_3_**], and diets supplemented with or without 25(OH)D_3_ on the fold change expression of muscle deposition-related genes in the pectoralis major muscle of broilers at 40 days of age (**doa**).

Treatment		N ^7^	MyoD1 ^1^	MyoG ^1^	Pax3 ^1^	Mrf-4 ^1^
*In ovo* injection						
	Non-injected ^2^	12	0.7 ^b^	1.0	0.7 ^b^	1.0
	MDV ^3^	12	0.8 ^b^	0.9	0.7 ^b^	1.4
	MDV + 25OHD_3_-1.2 ^4^	12	1.6 ^a^	1.7	2.8 ^a^	1.4
	MDV + 25OHD_3_-2.4 ^5^	12	1.7 ^a^	1.4	2.2 ^a^	1.1
	SEM		0.16	0.34	0.59	0.47
Diet						
	Commercial	24	0.9 ^b^	0.6 ^b^	0.8 ^b^	1.0
	Hy-D ^6^	24	1.5 ^a^	1.9 ^a^	2.3 ^a^	1.4
	SEM		0.22	0.25	0.44	0.33
			*p*-value			
*In ovo*			0.003	0.084	0.028	0.751
Diet			0.006	<0.001	0.013	0.209
*In ovo* x Diet			0.389	0.167	0.151	0.847

^a–b^ Treatment means within the same column within effect with no common superscripts are significantly different (*p* ≤ 0.05). ^1^ Myogenic differentiation proteins; myogenin; paired box domain 3; and myogenic regulatory transcription factor 4. ^2^ Embryos that were not injected with a solution. ^3^ Embryos injected with the commercial diluent in conjunction with Marek’s disease vaccine (**MDV**; turkey herpesvirus) at 18 d of incubation (**doi**). ^4^ Embryos injected with MDV containing 1.2 μg of 25OHD_3_ at 18 doi. ^5^ Embryos injected with MDV containing 2.4 μg of 25OHD_3_ at 18 doi. ^6^ A diet supplemented with 2760 IU/kg 25OHD_3_ throughout the rearing period. ^7^ Number of replications per treatment.

**Table 5:** Influence of noninjected; Marek’s disease vaccine (**MDV**)-injected; and MDV treatments containing various doses of calcidiol [**25(OH)D_3_**], and diets supplemented with or without 25(OH)D_3_ on the fold change expression of vitamin D activity-related genes in the pectoralis major muscle and spleen of broilers at 40 days of age (**doa**).

Treatment	N ^7^	CYP27B1 ^1^	CYP24A1 ^1^	VDR ^1^
		Breast		
*In ovo* injection				
Non-injected ^2^	12	0.8 ^b^	1.8 ^a^	1.0
MDV ^3^	12	0.9 ^b^	1.2 ^ab^	1.5
MDV + 25OHD_3_-1.2 ^4^	12	1.9 ^a^	0.9 ^b^	1.3
MDV + 25OHD_3_-2.4 ^5^	12	2.2 ^a^	0.7 ^b^	1.4
SEM		0.40	0.34	0.66
Diet				
Commercial	24	1.1 ^b^	1.5 ^a^	1.0
Hy-D ^6^	24	1.8 ^a^	0.8 ^b^	1.6
SEM		0.33	0.24	0.46
		*p*-value		
*In ovo*		0.050	0.017	0.897
Diet		<0.001	0.012	0.160
*In ovo* x Diet		0.114	0.433	0.534
		Spleen		
*In ovo* injection				
Non-injected	12	0.7	1.8 ^a^	1.1
MDV	12	1.0	2.2 ^a^	1.2
MDV + 25OHD_3_-1.2	12	1.8	0.7 ^b^	1.2
MDV + 25OHD_3_-2.4	12	1.6	0.8 ^b^	1.1
SEM		0.32	0.39	0.25
Diet				
Commercial	24	1.1	1.8 ^a^	1.2
Hy-D	24	1.4	1.0 ^b^	1.1
SEM		0.23	0.28	0.18
Diet x *in ovo* injection				
Commercial * Non-injected	6	0.9 ^bc^	2.6	1.4
Commercial * MDV	6	1.0 ^bc^	2.9	1.3
Commercial * MDV + 25OHD_3_-1.2	6	10 ^bc^	0.5	1.0
Commercial * MDV + 25OHD_3_-2.4	6	1.7 ^ab^	1.0	1.0
Hy-D * Non-injected	6	0.5 ^c^	1.1	0.7
Hy-D * MDV	6	1.1 ^bc^	1.5	1.1
Hy-D * MDV + 25OHD_3_-1.2	6	2.6 ^a^	0.9	1.5
Hy-D * MDV + 25OHD_3_-2.4	6	1.5 ^b^	0.6	1.1
SEM		0.36	0.58	0.35
*In ovo*		*p*-value		
Diet		0.005	0.001	0.852
*In ovo* x Diet		0.283	0.010	0.685
*In ovo* x Diet		0.010	0.065	0.124

^a–c^ Treatment means within the same column within effect with no common superscripts are significantly different (*p* ≤ 0.05). ^1^1α-hydroxylase; 24-hydroxylase; and vitamin D receptor. ^2^ Embryos that were not injected with a solution. ^3^ Embryos injected with the commercial diluent in conjunction with the Marek’s disease vaccine (**MDV**; turkey herpesvirus) at 18 d of incubation (**doi**). ^4^ Embryos injected with the MDV containing 1.2 μg of 25OHD_3_ at 18 doi. ^5^ Embryos injected with the MDV containing 2.4 μg of 25OHD_3_ at 18 doi. ^6^ A diet supplemented with 2760 IU/kg 25OHD_3_ throughout the rearing period. ^7^ Number of replications per treatment.

## Data Availability

The data of this study were not deposited in an official repository.

## References

[1] Zuidhof M.J., Schneider B.L., Carney V.L., Korver D.R., Robinson F.E. (2014). Growth, efficiency, and yield of commercial broilers from 1957, 1978, and 2005. Poult. Sci..

[2] Ferreira T.Z., Casagrande R.A., Vieira S.L., Driemerier D., Kindlein L. (2014). An investigation of a reported case of white striping in broilers. J. Appl. Poult. Res..

[3] Maiorano G. (2017). Meat defects and emergent muscle myopathies in broiler chickens: Implications for the modern poultry industry. Sci. Ann. Pol. Soc. Anim. Prod..

[4] Kuttappan V.A., Hargis B.M., Owens C.M. (2016). White striping and woody breast myopathies in the modern poultry industry: A review. Poult. Sci..

[5] Sihvo H.K., Immonen K., Puolanne E. (2014). Myodegeneration with fibrosis and regeneration in the Pectoralis major muscle of broilers. Vet. Pathol..

[6] Russo E., Drigo M., Longoni C., Pezzoti R., Fasoli P., Recordati C. (2015). Evaluation of white striping prevalence and predisposing factors in broilers at slaughter. Poult. Sci..

[7] Mutryn M.F., Brannick E.M., Fu W., Lee W.R., Abasht B. (2015). Characterization of a novel chicken muscle disorder through differential gene expression and pathway analysis using RNA-sequencing. BMC Genom..

[8] Bordini M., Soglia F., Davoli R., Zappaterra M., Petracci M., Meluzzi A. (2022). Molecular pathways and key genes associated with breast width and protein content in white striping and wooden breast chicken pectoral muscle. Front. Physiol..

[9] Williams C.J. (2011). *In ovo* vaccination and chick quality. Int. Hatch. Prac..

[10] Williams C.J. (2007). *In ovo* vaccination for disease prevention. Int. Poult. Prod..

[11] Das R., Mishra P., Jha R. (2021). *In ovo* feeding as a tool for improving performance and gut health of poultry: A review. Front. Vet. Sci..

[12] Peebles E.D. (2018). *In ovo* applications in poultry: A review. Poul. Sci..

[13] Uni Z., Ferket P.R., Tako E., Kedar O. (2005). *In ovo* feeding improves energy status of late-term chicken embryos. Poult. Sci..

[14] Uni Z., Ferket P.R. (2004). Methods for early nutrition and their potential. Worlds Poult. Sci. J..

[15] Fatemi S.A., Williams C.J., Deines J., Peebles E.D. (2023). Vitamin compatibility with the Marek’s disease vaccine. Poultry.

[16] Yair R., Shahar R., Uni Z. (2015). *In ovo* feeding with minerals and vitamin D_3_ improves bone properties in hatchlings and mature broilers. Poult. Sci..

[17] Fritts C.A., Waldroup P.W. (2003). Effect of source and level of vitamin D on live performance and bone development in growing broilers. J. Appl. Poult. Res..

[18] Xu H., Hu Z., Lu Y., Jiang Y., Li D., Lei B., Du R., Yang C., Zhang Z., Qiu M. (2023). Improvement in the early growth, immune system and tibia development of broilers in response to the *in ovo* injection of 25-hydroxyvitamin D_3_. J. Appl. Anim. Res..

[19] Ghobadi N., Hemati Matin H.R. (2017). The effect of *in ovo* injection of calcium, phosphorus, and vitamin-D on hatchability, blood biochemical parameters and bone of broiler chickens. Anim. Sci. J..

[20] Soares J.H., Kerr J.M., Gray R.W. (1995). 25-hydroxycholecalciferol in poultry nutrition. Poult. Sci..

[21] Yarger J.G., Quarles C.L., Hollis B.W., Gray R.W. (1995). Safety of 25-hydroxycholecalciferol as a source of cholecalciferol in poultry rations. Poult. Sci..

[22] Hutton K.C., Vaughn M.A., Litta G., Turner B.J., Starkey J.D. (2014). Effect of vitamin D status improvement with 25-hydroxycholecalciferol on skeletal muscle growth characteristics and satellite cell activity in broiler chickens. J. Anim. Sci..

[23] Fatemi S.A., Elliott K.E.C., Bello A., Durojaye O.A., Zhang H., Alqhtani A.H., Peebles E.D. (2021). Effects of the *in ovo* injection of vitamin D_3_ and 25-hydroxyvitamin D_3_ in Ross 708 broilers subsequently fed commercial or calcium and phosphorus-restricted diets. I. performance, carcass characteristics, and incidence of woody breast myopathy. Poult. Sci..

[24] Fatemi S.A., Elliott K.E.C., Bello A., Peebles E.D. (2021). Effects of the *in ovo* injection of vitamin D_3_ and 25-hydroxyvitamin D_3_ in Ross 708 broilers subsequently challenged with coccidiosis. I. performance, meat yield and intestinal lesion. Poult. Sci..

[25] Chou S.H., Chung T.K., Yu B. (2009). Effects of supplemental 25-hydroxycholecalciferol on growth performance, small intestinal morphology, and immune response of broiler chickens. Poult. Sci..

[26] Vignale K., Greene E.S., Caldas J.V., England J., Boonsinchai N., Sodsee P., Pollock E.D., Dridi S., Coon C.N. (2015). 25-Hydroxycholecalciferol enhances male broiler breast meat yield through the mTOR pathway. J. Nutr..

[27] Fatemi S.A., Elliott K.E.C., Bello A., Zhang H., Peebles E.D. (2021). Effects of the *in ovo* injection of vitamin D_3_ and 25-hydroxyvitamin D_3_ in Ross 708 broilers subsequently fed commercial or calcium and phosphorous-restricted diets: II. Immunity and small intestine morphology. Poult. Sci..

[28] Fatemi S.A., Macklin K.S., Zhang L., Mousstaaid A., Poudel S., Poudel I., Peebles E.D. (2022). Improvement in the immunity- and vitamin D_3_ activity-related gene expression of coccidiosis-challenged Ross 708 broilers in response to the *in ovo* injection of 25-hydroxyvitamin D_3_. Animals.

[29] Fatemi S.A., Elliott K.E.C., Bello A., Macklin K.S., Peebles E.D. (2022). Effects of the *in ovo* injection of vitamin D_3_ and 25-hydroxyvitamin D_3_ in Ross 708 broilers subsequently challenged with coccidiosis: II. Immunological and inflammatory responses and small intestine histomorphology. Animals.

[30] Shojadoost B., Behboudi S., Villanueva A.I., Brisbin J.T., Ashkar A.A., Sharif S. (2015). Vitamin D_3_ modulates the function of chicken macrophages. Res. Vet. Sci..

[31] Morris A., Shanmugasundaram R., Lilburn M.S., Selvaraj R.K. (2014). 25-Hydroxycholecalciferol supplementation improves growth performance and decreases inflammation during an experimental lipopolysaccharide injection. Poult. Sci..

[32] Morris A., Selvaraj R.K. (2014). In vitro 25-hydroxycholecalciferol treatment of lipopolysaccharide-stimulated chicken macrophages increases nitric oxide production and mRNA of interleukin-1 beta and 10. Vet. Immunol. Immunopathol..

[33] Morris A., Shanmugasundaram R., McDonald J., Selvaraj R.K. (2015). Effect of in vitro and in vivo 25-hydroxyvitamin D treatment on macrophages, T cells, and layer chickens. J. Anim. Sci..

[34] Shanmugasundaram R., Morris A., Selvaraj R.K. (2019). Effect of 25-hydroxycholecalciferol supplementation on turkey performance and immune cell parameters in a coccidial infection model. Poult. Sci..

[35] Nong K., Liu Y., Fang X., Qin X., Liu Z., and Zhang H. (2023). Effects of the vitamin D_3_ on alleviating the oxidative stress induced by Diquat in Wenchang chickens. Animals.

[36] Ding B., Pirone A., Lenzi A. (2011). Baglini, and I. Romboli. 2011. Effect of hen diet supplemented with 25-OH-D_3_ on the development of small intestinal morphology of chick. J. Anim. Feed. Sci..

[37] Smith J.E., Goodman D.S. (1971). The turnover and transport of vitamin D and of a polar metabolite with the properties of 25-hydroxycholecalciferol in human plasma. J. Clin. Investig..

[38] Hollis B.W., Wagner C.L. (2013). Clinical review: The role of the parent compound vitamin D with respect to metabolism and function: Why clinical dose intervals can affect clinical outcomes. J. Clin. Endocrinol. Metab..

[39] Fatemi S.A., Mousstaaid A., Williams C.J., Deines J., Poudel S., Poudel I., Elliott K.E.C., Walters E.R., Forcier N., Peebles E.D. (2024). *In ovo* administration of the Marek’s Disease vaccine in conjunction with 25-hydroxyvitamin D_3_ and its subsequent effects on the performance and immunity-related characteristics of Ross 708 broiler hatchlings. Poult. Sci..

[40] Fatemi S.A., Levy A.W., Peebles E.D. (2024). Enhancements in the expressions of genes associated with the immunity, muscle growth, and antioxidant activity of 14 d broilers in response to the *in ovo* injection of the Marek’s Disease vaccine alone or in conjunction with the *in ovo* and dietary supplemental administration of 25-hydroxycholecalciferol. Poult. Sci..

[41] Fatemi S.A., Mousstaaid A., Williams C.J., Deines J., Poudel S., Poudel I., Walters E.R., Levy A.W., Peebles E.D. (2024). Effects of the *in ovo* administration of the Marek’s Disease Vaccine alone or in combination with the supplemental *in ovo* and dietary administration of 25-hydroxyvitamin D_3_ on the performance, meat yield, and incidence of woody breast myopathy in Ross 708 broilers. Animals.

[42] Fatemi S.A., Levy A.W., Peebles E.D. (2024). Ross 708 broiler small intestine morphology and immunity improvements in response to *in ovo* Marek’s Disease vaccine administration alone or in conjunction with *in ovo* and dietary supplemental cholecalciferol. Poult. Sci..

[43] Aviagen (2015). Ross 708 Pocket Guide.

[44] Karpala A.J., Bagnaud-Baule A., Goossens K.E., Lowenthal J.W., Bean A.G. (2012). Ontogeny of the interferon system in chickens. J. Reprod. Immunol..

[45] Gimeno I.M., Faiz N.M., Cortes A.L., Barbosa T., Villalobos T., Pandiri A.R. (2015). *In ovo* vaccination with turkey herpesvirus hastens maturation of chicken embryo immune responses in specific-pathogen-free chickens. Avian Dis..

[46] Tang D.J., Wu H., Jiao X., Wang J., Zhao Lin H. (2019). The development of antioxidant system in the intestinal tract of broiler chickens. Poult. Sci..

[47] Yin H., Zhang S., Gilbert E.R., Siegel P.B., Zhu Q., Wong E.A. (2014). Expression profiles of muscle genes in postnatal skeletal muscle in lines of chickens divergently selected for high and low body weight. Poult. Sci..

[48] SAS Institute (2013). SAS Proprietary Software Release 9.4.

[49] Steel R.G.D., Torrie J.H. (1980). Principles and Procedures of Statistics: A Biometrical Approach.

[50] Stewart C.R., Bagnaud-Baule A., Karpala A.J., Lowther S., Mohr P.G., Wise T.G., Lowenthal J.W., Bean A.G. (2012). Toll-like receptor 7 ligands inhibit influenza A infection in chickens. J. Interferon Cytokine Res..

[51] Lu Y., Sarson A.J., Gong J., Zhou H., Zhu W., Kang Z., Yu H., Sharif S., Han Y. (2009). Expression profiles of genes in Toll-like receptor-mediated signaling of broilers infected with *Clostridium perfringens*. Clin. Vaccine Immunol..

[52] Nawab A., An L., Wu J., Li G., Liu W., Zhao Y., Wu Q., Xiao M. (2019). Chicken toll-like receptors and their significance in immune response and disease resistance. Int. Rev. Immunol..

[53] Wigley P., Kaiser P. (2003). Avian cytokines in health and disease. Braz. J. Poult. Sci..

[54] Wlaźlak S., Pietrzak E., Biesek J., Dunislawska A. (2023). Modulation of the immune system of chickens a key factor in maintaining poultry production-a review. Poult. Sci.

[55] Wan Y.Y., Flavell R.A. (2007). ‘Yin-Yang’ functions of transforming growth factor-beta and T regulatory cells in immune regulation. Immunol. Rev..

[56] Sojka D.K., Huang Y.H., Fowell D.J. (2008). Mechanisms of regulatory T-cell suppression—A diverse arsenal for a moving target. Immunology.

[57] Shanmugasundaram R., Selvaraj R.K. (2011). Regulatory T cell properties of chicken CD4+CD25+ cells. J. Immunol..

[58] Lowenthal J.W., Lambrecht B., van den Berg T.P., Andrew M.E., Strom A.D., Bean A.G. (2000). Avian cytokines—The natural approach to therapeutics. Dev. Comp. Immunol..

[59] Gimeno I.M., Glaize A., Cortes A.L. (2018). Effect of Marek’s disease vaccines on interferon and toll like receptors when administered *in ovo*. Vet. Immunol. Immunopathol..

[60] Zhang L., Tai Y.T., Ho M.Z.G., Qiu L., Anderson K.C. (2017). Interferon-alpha-based immunotherapies in the treatment of B cell-derived hematologic neoplasms in today’s treat-to-target era. Exp. Hematol. Oncol..

[61] Layton D.S., Mara K., Dai M., Malaver-Ortega L.F., Gough T.J., Bruce K., Jenkins K.A., Bean A.G.D. (2022). Interferon signaling in chickens plays a crucial role in inhibiting influenza replication in DF1 cells. Microorganisms.

[62] Bar-Shira E., Friedman A. (2006). 2006. Development and adaptations of innate immunity in the gastrointestinal tract of the newly. hatched chick. Dev. Comp. Immunol..

[63] Mavrommatis A., Simitzis P.E., Kyriakaki P., Giamouri E., Myrtsi E.D., Evergetis E., Filippi K., Papapostolou H., Koulocheri S.D., Pappas A.C. (2021). Immune-related gene expression profiling of broiler chickens fed diets supplemented with vinification byproducts: A valorization approach II. Animals.

[64] Li B., Kalmu N., Dong X., Zhang Y., Puolanne E., Ertbjerg P. (2024). Relationship between wooden breast severity in broiler chicken, antioxidant enzyme activity and markers of energy metabolism. Poult. Sci..

[65] Surai P.F., Kochish I.I., Fisinin V.I., Kidd M.T. (2019). Antioxidant defence systems and oxidative stress in poultry biology: An update. Antioxidants.

[66] Surai P.F. (2016). Antioxidant systems in poultry biology: Superoxide dismutase. J. Anim. Res. Nutr..

[67] Brieger K., Schiavone S., Miller F.J., Krause K.H. (2012). Reactive oxygen species: From health to disease. Swiss Med. Wkly..

[68] Surai P., Kochish I., Fisinin V. (2018). Glutathione peroxidases in poultry biology: Part 1. Classification and mechanisms of action. Worlds Poult. Sci. J..

[69] Pan X., Zhang L., Xing T., Li J., Gao F. (2021). The impaired redox status and activated nuclear factor-erythroid 2-related factor 2/antioxidant response element pathway in wooden breast myopathy in broiler chickens. Anim. Biosci..

[70] Hisasaga C., Makagon M.M. (2024). Increased activity reduces the prevalence of woody breast in Ross 708 and Ranger Gold broilers. Poult. Sci..

[71] Tavleeva M.M., Belykh E.S., Rybak A.V., Rasova E.E., Chernykh A.A., Ismailov Z.B., Velegzhaninov I.O. (2022). Effects of Antioxidant gene overexpression on stress resistance and malignization in vitro and in vivo: A review. Antioxidants.

[72] Yang L., Liu G., Zhu X., Luo Y., Shang Y., Gu X.L. (2019). The anti-inflammatory and antioxidant effects of leonurine hydrochloride after lipopolysaccharide challenge in broiler chicks. Poult. Sci..

[73] Chen C., White D.L., Marshall B., Kim W.K. (2021). Role of 25-Hydroxyvitamin D_3_ and 1,25-Dihydroxyvitamin D_3_ in chicken embryo osteogenesis, adipogenesis, myogenesis, and vitamin D_3_ metabolism. Front. Physiol..

[74] Liu Q.C., Zha X.H., Faralli H., Yin H., Louis-Jeune C., Perdiguero E., Pranckeviciene E., Muñoz-Cànoves P., Rudnicki M.A., Brand M. (2012). Comparative expression profiling identifies differential roles for Myogenin and p38α MAPK signaling in myogenesis. J. Mol. Cell Biol..

[75] Yablonka-Reuveni Z., Paterson B.M. (2001). MyoD and myogenin expression patterns in cultures of fetal and adult chicken myoblasts. J. Mol. Histol..

[76] Mok G.F., Mohammed R.H., Sweetman D. (2015). Expression of myogenic regulatory factors in chicken embryos during somite and limb development. J. Anat..

[77] Haldar M., Karan G., Tvrdik P., Capecchi M.R. (2008). Two cell lineages, myf5 and myf5-independent, participate in mouse skeletal myogenesis. Dev. cell.

[78] Moncaut N., Rigby P.W., Carvajal J.J. (2013). Dial M(RF) for myogenesis. FEBS J..

[79] Genxi Z., Ying T., Tao Z., Jinyu W., Yongjuan W. (2014). Expression profiles and association analysis with growth traits of the MyoG and Myf5 genes in the Jinghai yellow chicken. Mol. Biol. Rep..

[80] Daughtry M.R., Berio E., Shen Z., Suess E.J.R., Shah N., Geiger A.E., Berguson E.R., Dalloul R.A., Persia M.E., Shi H. (2017). Satellite cell-mediated breast muscle regeneration decreases with broiler size. Poult. Sci..

[81] Otto A., Schmidt C., Patel K. (2006). Pax3 and Pax7 expression and regulation in the avian embryo. Anat. Embryol.

[82] Shinin V., Gayraud-Morel B., Gomès D., Tajbakhsh S. (2006). Asymmetric division and cosegregation of template DNA strands in adult muscle satellite cells. Nat. Cell Biol..

[83] Day K., Shefer G., Richardson J.B., Enikolopov G., Yablonka-Reuveni Z. (2007). Nestin-GFP reporter expression defines the quiescent state of skeletal muscle satellite cells. Dev. Biol..

[84] Clark D.L., Coy C.S., Strasburg G.M., Reed K.M., Velleman S.G. (2016). Temperature effect on proliferation and differentiation of satellite cells from turkeys with different growth rates. Poult. Sci..

[85] Norman A.W. (1987). Studies on the vitamin D endocrine system in the avian. J. Nutr..

[86] Pfotenhauer K.M., Shubrook J.H. (2017). Vitamin D deficiency, its role in health and disease, and current supplementation recommendations. J. Am. Osteopath. Assoc..

[87] Shanmugasundaram R., Selvaraj R.K. (2012). Vitamin D-1alpha-hydroxylase and vitamin D-24-hydroxylase mRNA studies in chickens. Poult. Sci..

[88] de Matos R. (2008). Calcium metabolism in birds. Vet. Clin. North. Am. Exot. Anim. Pract..

[89] Dzik K.P., Kaczor J.J. (2019). Mechanisms of vitamin D on skeletal muscle function: Oxidative stress, energy metabolism and anabolic state. Eur. J. Appl. Physiol..

